# Educational Needs of Adolescents Regarding Normal Puberty and Menstrual Patterns

**DOI:** 10.4274/jcrpe.2144

**Published:** 2015-12-03

**Authors:** Pınar İşgüven, Göze Yörük, Filiz Mine Çizmecioğlu

**Affiliations:** 1 Sakarya University Faculty of Medicine, Department of Pediatric Endocrinology, Sakarya, Turkey; 2 Medeniyet University Faculty of Medicine, Göztepe Research and Education Hospital, Clinic of Pediatrics, İstanbul, Turkey; 3 Kocaeli University Faculty of Medicine, Department of Pediatric Endocrinology, Kocaeli, Turkey

**Keywords:** Normal puberty, menstrual pattern, puberty education, adolescent school girls

## Abstract

**Objective:**

The study aimed to determine the level of knowledge and the sources of information about normal puberty and menstrual patterns in Turkish schoolgirls from İstanbul.

**Methods:**

The study sample was comprised of 922 randomly chosen schoolgirls. A questionnaire survey of knowledge of normal pubertal development and menstrual patterns was conducted.

**Results:**

The age of the girls ranged between 10 and 17 years and 82.3% had had menarche. The leading source of pubertal information was the mothers (84.2%). There was no statistically significant relationship between the mothers’ education level and the level of knowledge of the students about pubertal development (p>0.05). The main source for 18% of students was their teacher, but only 6% had a preference for their teacher providing education on this topic. Students who attained menarche preferred education about puberty to be given by health professionals and to both genders at the same setting (p<0.01). A total of 31.5% of students thought that the first symptom of puberty was acne. Half (50.7%) of the students did not know the time period between the beginning of puberty and menarche. The girls who had attained menarche were more knowledgeable about puberty, largely through their own experience.

**Conclusion:**

This study shows that schoolgirls have an insufficient level of knowledge about normal puberty. Education programs must be conducted for students and their parents.

**WHAT IS ALREADY KNOWN ON THIS TOPIC?**

Concerns about the normality of pubertal development and of menstrual patterns are among the most common problems of young girls. Girls frequently have difficulty assessing what represents normal pubertal development and menstrual cycle, or patterns of bleeding.

**WHAT THIS STUDY ADDS?**

The findings in this study show that in Turkey, there is a need to establish school-based reproductive health education programs to enable schoolgirls to learn how to cope with these critical issues.

## INTRODUCTION

Concerns about the normality of pubertal development and of menstrual patterns are among the most common problems of young girls (1). Girls frequently have difficulty assessing what represents normal pubertal development and menstrual cycle, or patterns of bleeding (2). With these concerns, girls may refrain from informing their parents about their menstrual irregularities and missed menses. On the other hand, these same findings may indicate dysregulation of the hypothalamic-pituitary-ovarian (HPO) axis. A thorough evaluation of menstrual cycle disorders in adolescence provides an opportunity to diagnose and treat conditions affecting the HPO axis in a timely manner (3).

In this study, we investigated the level of knowledge on normal pubertal development in schoolgirls attending primary and secondary schools in a central district of İstanbul, the sources from which they obtain this information and their expectations regarding education about sexual development.

## METHODS

This was a cross-sectional study on a school-based sample of Turkish girls aged 10-17 years attending primary and secondary schools. Between April and June 2012, female students from two primary and three randomly selected secondary schools in the Göztepe district of İstanbul were invited to participate in the study.

The study has been performed with the permission of the local Ethics Committee. Written permission was also obtained from the Ministry of Education. After provision of information about the aims of the study, written informed consent was obtained from the students and their parents.

The data were collected by the questionnaire method. All the schools included in the study were coeducational. The 1,000 questionnaires given out were more than the required sample size of 460; this was to ensure that a sufficient number of completed questionnaires were returned for our data analysis.

The relevance of the study was explained to the female students before handing out the questionnaires. Male students were sent out of the classroom while the girls were filling out the questionnaires. We also explained that their personal information would be kept confidential and that all the data would only be used for research purposes.

The questionnaires included 15 multiple choice questions that allowed students to choose more than one option for some of the questions. The questionnaire was developed by researchers based on the current knowledge on the subject. The questionnaire used had been pretested in a similar group of girls at a different site to ensure that it was possible to self-administer it in the target group. All unknown terminologies (i.e., puberty, menarche) were described and explained before the girls started to complete the questionnaire. The questionnaire consisted of 2 parts. Part 1 included questions about socio-demographic information such as current age, parents’ education level, how they obtained information regarding puberty, whether the responder had attained menarche and, if so, the age at menarche. Part 2 consisted of questions aiming to investigate their knowledge about normal pubertal development. The subjects were asked questions about the first symptom of puberty, the normal average time period between the onset of puberty and menarche, the time span of a normal menstrual cycle, age of menarche, the average duration of bleeding, and the normal number for changes of pads per day without taking into consideration their own experience. A sample blank questionnaire is shown in Appendix A.

The girls were asked to indicate their birth date (day, month, year) and the date of their first menstrual bleeding as accurately as possible (at least the month and the year); when information on the day was missing, the event was considered to have happened at mid-month. Age at the time of the questionnaire and age at menarche were expressed as decimal years. The definitions used to describe normal puberty and menstrual cycle are shown below (4,5,6):

- Breast development: 8-13 years,

- The time period from breast stage 2 to menarche: 2-4 years,

- Mean menarche age: 12-13 years,

- Mean cycle interval: 21-45 days,

- Menstrual flow length: ≤7 days,

- Daily use of pads during menstruation: 3-6 pads.

The students were categorized into two groups according to their self-identification of knowledge about puberty. Group 1 consisted of students who believed that their knowledge about puberty was sufficient. Group II stated that they had an insufficient level of knowledge.

### Statistical Analysis

Number Cruncher Statistical System 2007&Power Analysis and Sample Size 2008 Statistical Software (Utah, USA) program were used for statistical analyses. When evaluating study data, Pearson chi-squared test, Fisher’s exact test, and Yates continuity correction test (Yates corrected chi-squared) were used to compare qualitative data in addition to defining statistical methods (mean, standard deviation, frequency, ratio). P-values <0.01 and <0.05 were considered to be statistically significant.

## RESULTS

Of the 1000 questionnaires dispensed to the students, 78 were excluded from the survey due to not reporting critical data items such as their exact dates of birth or age at menarche. The remaining 85% (922 girls) of the original sample constituted the study group. A total of 36.8% (n=339) subjects were primary school students (5th, 6th, 7th, 8th grades) and 63.2% (n=583) were secondary school students (9th, 10th, 11th grades). The age of the subjects ranged between 10 and 17 years with a mean of 14.7±2.0. Of the girls, 82.3% (n=759) had attained menarche by the time the survey was conducted. The age of menarche ranged between 10-16 years and the mean age was 12.9±1.2 years.

The questionnaire results revealed that 7.2% (n=66) of mothers were illiterate, 67.6% (n=623) were primary school graduates, and 25.3% (n=233) were secondary school or university graduates. Similar data for fathers’ education level showed that 3.9% (n=36) were illiterate, 61.8% (n=570) were primary school graduates, and 34.3% (n=316) were secondary school or university graduates. There was no statistically significant relationship between the mother’s education level and the level of knowledge of the student about pubertal development (p>0.05). However, the proportion of students whose mothers were secondary school or university graduates who knew the length of a normal menstrual cycle was significantly higher.

Of the students, 75.2% (n=693) thought they had a sufficient level of knowledge about puberty, while 84.2% (n=776) stated that they had obtained their first information from their mothers. Only 0.8% (n=7) cited the father as the source. Some students had received information from multiple resources (Table 1).

A total of 31.5% (n=290) of students mistakenly thought that the first symptom of puberty was facial acne. Students who thought that puberty started with breast development were 23.9% (n=220) of the total study group, while 23.4% (n=216) thought that it started with getting pubic and axillary hair. While half of the students did not know the time period between the beginning of puberty and menarche, only 13.6% (n=125) knew it correctly. The majority of the students had accurate knowledge about menstrual cycle characteristics (Table 2).

There was no statistical difference between the students who self-identified as being knowledgeable about puberty and those who did not, in terms of knowing the correct time period between the first symptom of puberty and menarche (p>0.05). Moreover, students who self-identified as being knowledgeable thought that puberty started with getting pubic and axillary hair (p<0.05) (Table 3).

The knowledge on normal age for attaining menarche was correct in more than half of the students, regardless of their self-knowledge. Group 1, who thought they had a sufficient knowledge about puberty, had a better knowledge of menstrual cycle length, menstrual flow duration, and daily pad use compared to the other group (Table 4).

Eighty percent of the students (n=607) who self-identified as being knowledgeable about puberty had already attained menarche. Regardless of menarche, most of the students (menarche (+) group -76.4%; menarche (-) group -71.8%) preferred to be taught about puberty in female-only classes. Students who had attained menarche had a higher preference for education about puberty to be given by health professionals and to both genders at the same setting as compared to those who were premenarcheal (p<0.01) (Table 5).

There was no correlation between knowing the normal age of menarche and the student’s status of menarche (p>0.05). Students who had attained menarche had a significantly higher ratio of stating the first symptom of puberty as pubic and axillary hair (p<0.01). The students who had attained menarche correctly answered the questions about the time period between beginning of puberty and menarche, cycle length, menstrual flow duration, and daily pad use (p<0.01) (Table 6).

## DISCUSSION

The results show that not only was knowledge regarding puberty poor among the subjects, but also their knowledge regarding what constitutes a normal menstrual cycle was not satisfactory. The findings suggest that health and education authorities need to recognize the problem and cooperate to provide appropriate support for schoolgirls in the school environment.

İstanbul is the biggest city in Turkey and the population is quite representative for the whole country because of emigration from all parts of the country. Our study took place in central Istanbul and the average age of menarche was found to be 12.9 years. Although there is a debate about secular trends in puberty worldwide, our national reference data have shown that the age of menarche has not changed over the last 40 years (7,8,9,10,11). Also, age of menarche did not differ greatly from that in European countries or the USA (12). It is worth noting that the aim of this study was not to determine the average age of menarche in the country. Menarche is an important event in the life of girls. Since menarche is the last phase of puberty, age of menarche should not be the point at which pubertal education is given to the students. In Turkish girls, the onset of puberty begins at around 9 years (9), therefore, we believe this education should start around 8-9 years of age before the first signs of puberty are apparent.

A great majority of the 922 schoolgirls who participated in our study stated that they obtained their first information about puberty from their mothers (84%). On the other hand, we found that there is almost no dialogue between fathers and daughters (0.8%). This finding is in line with other studies which show that girls often feel uncomfortable talking about menstruation with their fathers (13,14,15). While one in five girls discussed pubertal issues with their friends, 7% obtained information from the media and the internet. Since getting information from the media, the internet, and friends may lead to misinformation, the low numbers may be interpreted as a positive finding. On the other hand, the widespread use of the internet may be a feasible opportunity to gain access in privacy to a program of pubertal and sexual education prepared by educational and health professionals in collaboration. The main source for 18% of the students was their teacher, but only 6% had a preference for their teacher providing education on this topic. This suggests that this aspect of the education system should be reconstructed on a level that children can relate to and in a way that addresses their needs.

Our findings show that 7% of mothers were illiterate, while 67.6% were primary school graduates. The correct answers the students gave to questions about pubertal physiology and menstruation were not related to the level of education of the mothers. On the other hand, students whose mothers were secondary school or university graduates had a more accurate knowledge of the menstrual cycle. Similar to our findings, Demirel and Terzioglu (15) found that mothers’ level of education had no effect on the level of knowledge of the student on pubertal physiology. Erbil et al (16) reported that 60.8% of mothers informed their daughters about pubertal matters; however, 65.6% of these mothers had never talked to their own mothers about sexual matters. The fact that students who, for the most part, obtained their education from their mothers had an insufficient level of knowledge, and the lack of a significant relationship between the mothers’ level of education and obtaining correct information as a child both showed that the mothers were not knowledgeable enough on the subject matter. It appears that a major source of knowledge about pubertal development and menstruation is personal experience which is then refined by discussion with mothers and friends.

It has been shown that even in as developed a country as the USA these issues are “taboo”. In closed societies like the Hmong, it has been shown that mothers do speak with their daughters about menstruation and puberty but instead of providing accurate information, give incorrect information, often based on fear of menstruation (17). This type of incorrect information may cause the children to go through the pubertal period under pressure, be ashamed of the changes happening to their body.

The main reasons for the majority of the students having insufficient and inaccurate knowledge about puberty are inadequate sources of information within the family and the taboo to talk about sexuality still existing in the Turkish society. In this day and age, it will be helpful for health professionals to actively participate in education programs in schools. In the questionnaire, 54% of the children already stated that they would prefer education about puberty to be provided by health professionals. A study from Iran indicates that school health trainers play an essential role in knowledge transfer, attitude promotion of adolescent girls (18).

Female-only school classes for education pertaining to puberty and menstrual cycles is preferred by 75% of the participants and this preference does not change according to the status of menarche. However, students who have attained menarche are significantly more positive about getting pubertal education in mixed gender classes. Attaining menarche significantly decreases the number of students who think education is not necessary or state that they do not know. With the beginning of menstrual cycles, there is a change in the attitude of the students and they are more interested in education on this topic. In addition to wanting to learn about their male peers’ pubertal development, they want the other gender to know and share their own pubertal changes.

On the topic of pubertal period and menstrual cycle, 75% of the students thought they had a sufficient level of knowledge. Upon investigating the level of knowledge about first pubertal symptoms, only 24% identified breast development, while 23% chose pubic and axillary hair. Having acne on the face was thought to be the correct answer by 32%. Students identifying acne on the face as the first sign of puberty may be related to how much they care about their appearance due to their age and the fact that they are sensitive about beauty. Development of breast tissue is generally considered to be representative of the pubertal reactivation of the hypothalamic-pituitary-gonadal axis in girls; many studies as well as clinicians evaluate the onset of breast development as the onset of puberty in girls (2,4,7,9,19,20,21). Although the onset of breast development characteristically precedes the appearance of pubic hair, there is normally considerable variation in the sequence of these events. Pubic hair may appear before breast budding, usually due to a lack of direct linkage between adrenarche and gonadal development. Ibanez et al (22) has questioned whether the appearance of pubic hair without breast development represents true pubertal maturation, or if it is merely a manifestation of adrenarche or premature adrenarche. Biro et al (23) suggested that development of pubic hair without breast development may represent true pubertal maturation in girls. A Danish study showed only 6.8% of the girls entered puberty by the pubarche pathway (24), whereas the prevalence of girls entering puberty by the pubarche pathway was 11.6% in a UK study and 14.5% in a USA study (25,26). In our society, the proportion of having pubic and axillary hair before breast development has been reported to be 21.5% (9). Therefore, the appearance of pubic and axillary hair might also be evaluated as an acceptable answer as the first symptom of puberty. However, we need a greater number of longitudinal studies on this debatable topic.

There was no statistical significance regarding correct answers to questions about the pubertal development process between students who thought they were knowledgeable about pubertal development and the menstrual cycle and those who did not think they were knowledgeable. However, it was observed that students who had attained menarche had a greater percentage of correct answers regarding the menstrual cycle than those who did not. All these results show that students were significantly influenced by their own experience regarding their knowledge about the menstrual cycle.

Why is it important to have accurate knowledge about normal menstrual cycles? Two large studies, one cataloging 275,947 cycles in 2,702 females and another reporting on 31,645 cycles in 656 females, support the observation that menstrual cycles in girls and adolescents typically range from 21 to 45 days (27,28). The 95th percentile for menstrual cycle length is 90 days, thus secondary amenorrhea should be defined by this evidence-based criterion as 90 days (4). Dewhurst et al (29) analyzed 368 menstrual periods and found that the flow lasted between 3 and 7 days in 88% of the cycles, with an average length of 5 days. In another study on menstrual patterns among Italian adolescent girls, Rigon et al (30) reported that a shorter than normal bleeding period (<4 days) was reported by 3.2% of their sample population and a long bleeding period (>7 days) by 19% of the girls. Most of the participants in our study had correct knowledge about mean cycle interval, menstrual flow length, and daily pad use. Having the correct knowledge of normal cycle length, menstrual period duration, and the amount of bleeding will inform and prevent complications caused by dysfunctional uterine bleedings in the first years after attaining menarche. In Lebanon, Karout et al (31) reported that around half of the students in their study had polymenorrhoea. Prolonged menstrual bleeding can result in iron deficiency anemia. Menstrual dysfunction can have significant effects on daily activities and result in poor school performance. An Australian study showed that approximately 25% of girls had severe menstrual dysfunction affecting daily activities and resulting in school absence (32). Santina et al (33) reported that of the 389 post-menarcheal schoolgirls, 35.2% had irregular cycles and 74.3% had dysmenorrhea. Adolescents with cycles that are consistently outside of the range of 21-45 days should be evaluated for pathologic conditions such as polycystic ovary syndrome, eating disorders, thyroid disease, hyperprolactinemia, or even ovarian insufficiency. We recommend that adolescents be encouraged to chart their menstrual frequency and regularity from menarche to adulthood. Only upon knowing the normal pattern of their own menstrual cycle can adolescents notice a potentially pathological change and ask for medical help in a timely manner. For all these reasons, adolescents having correct information about normal menstrual patterns outside of their own experience would allow problems on this subject to be treated before they reach an advanced stage.

In conclusion, the findings in this study show that in Turkey, there is a need to establish school-based reproductive health education programs to enable schoolgirls to learn how to cope with these critical issues. Education about puberty at the level the students can comprehend and which will address their needs should be provided in schools. This should start at young ages, before first signs of puberty are apparent. The support and involvement of health care professionals in this education would be welcomed by girls attending these classes. Since the first target in education about puberty is the mother, a family education program should be constructed that is suited to our culture.

## Figures and Tables

**Appendix A.**
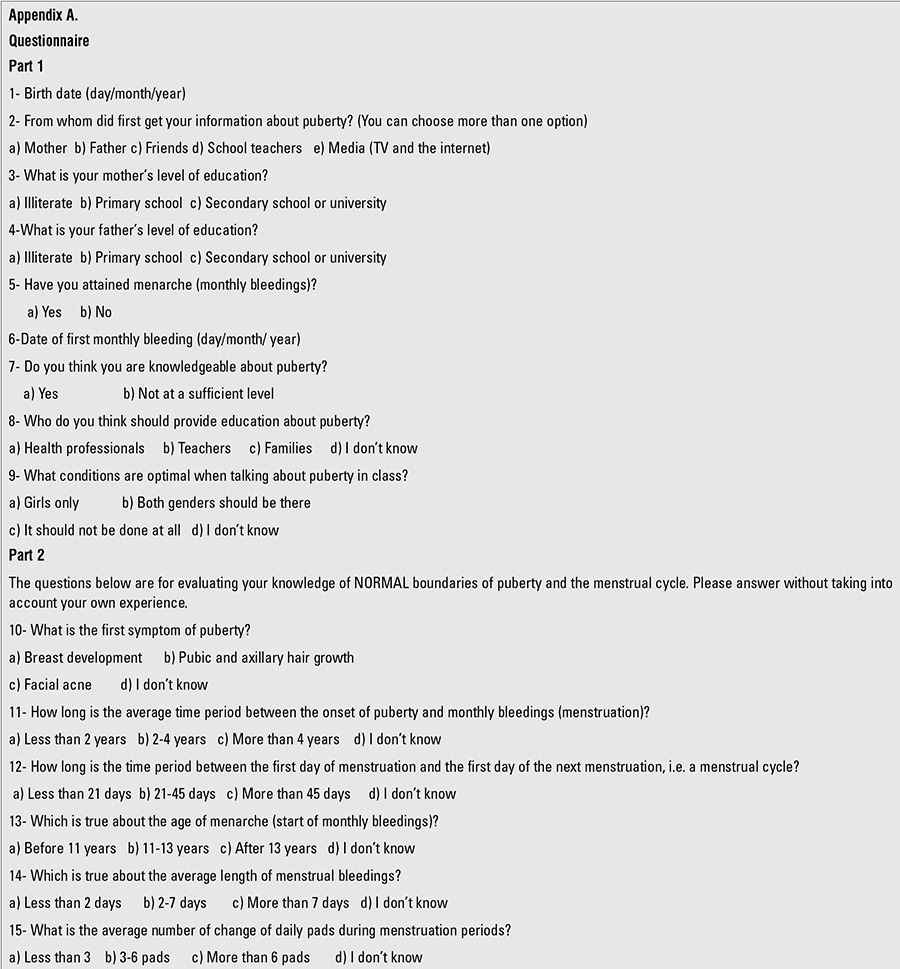
Appendix A.

**Table 1:**
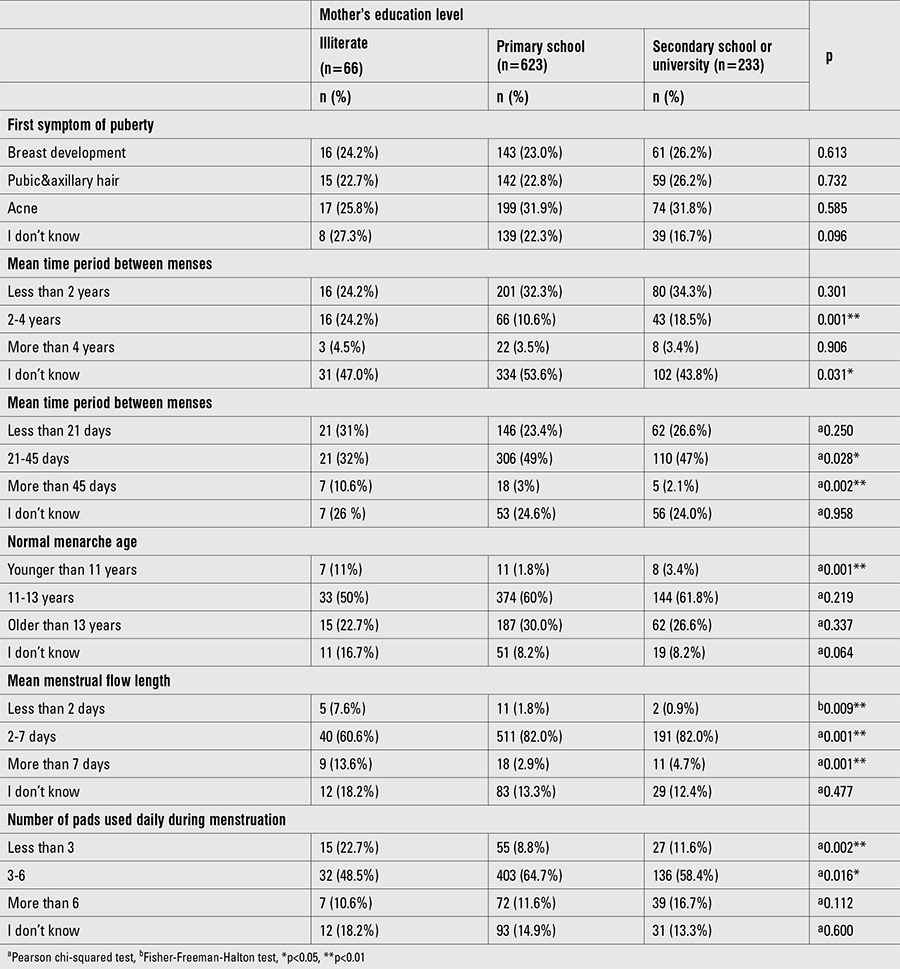
Puberty and menstrual cycle knowledge by the mother’s education level

**Table 2:**
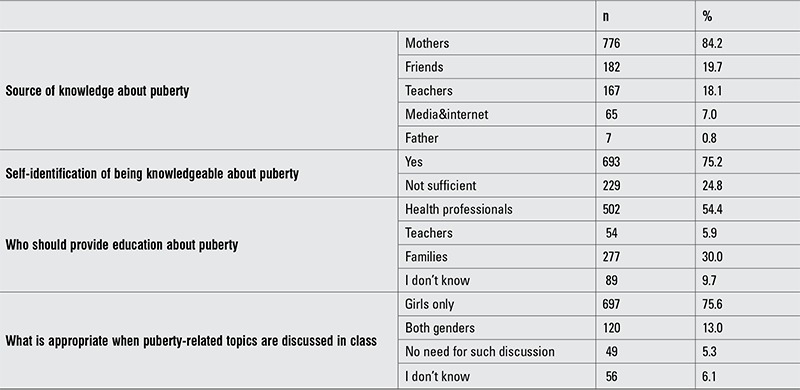
Source of knowledge about puberty, self-evaluation and opinions on education about puberty

**Table 3:**
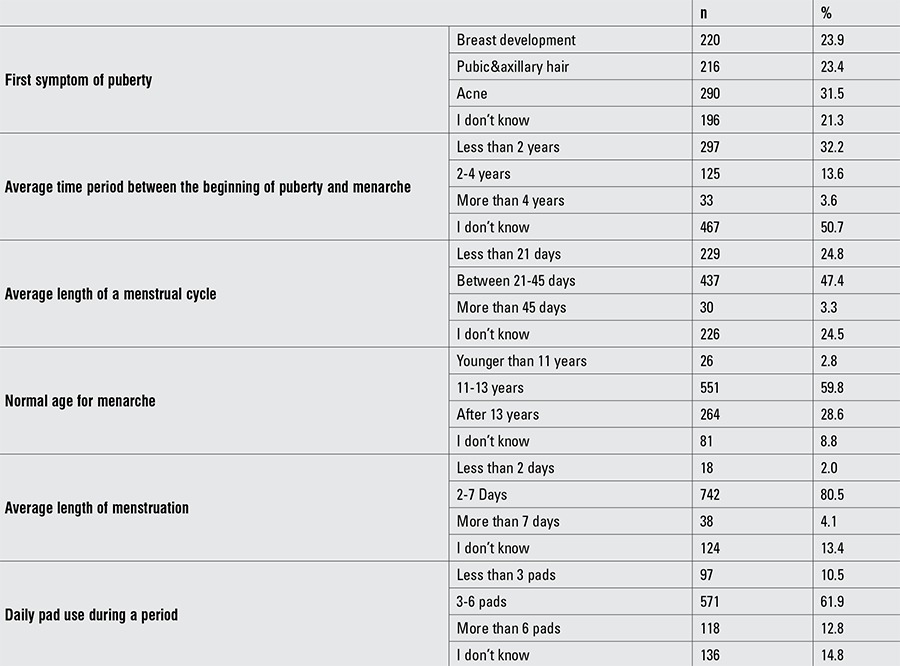
Knowledge on normal puberty characteristics and on menstrual cycle

**Table 4:**
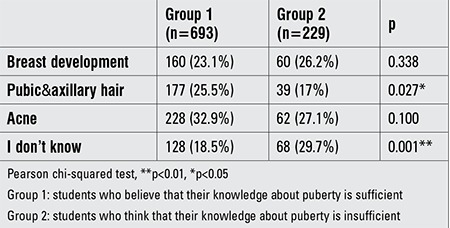
First sign of puberty stated by group 1 and group 2 students

**Table 5:**
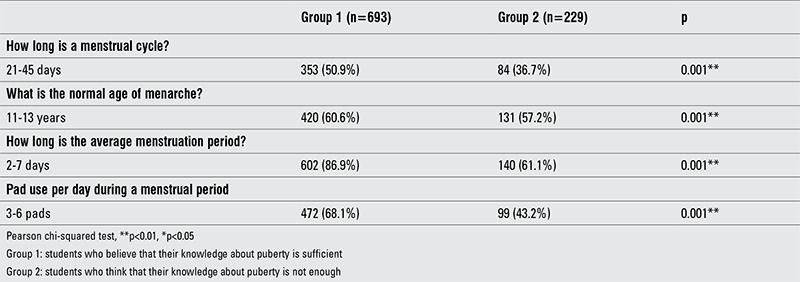
Knowledge about menarche and the normal pattern of menstrual cycles in group 1 and group 2 students

**Table 6:**
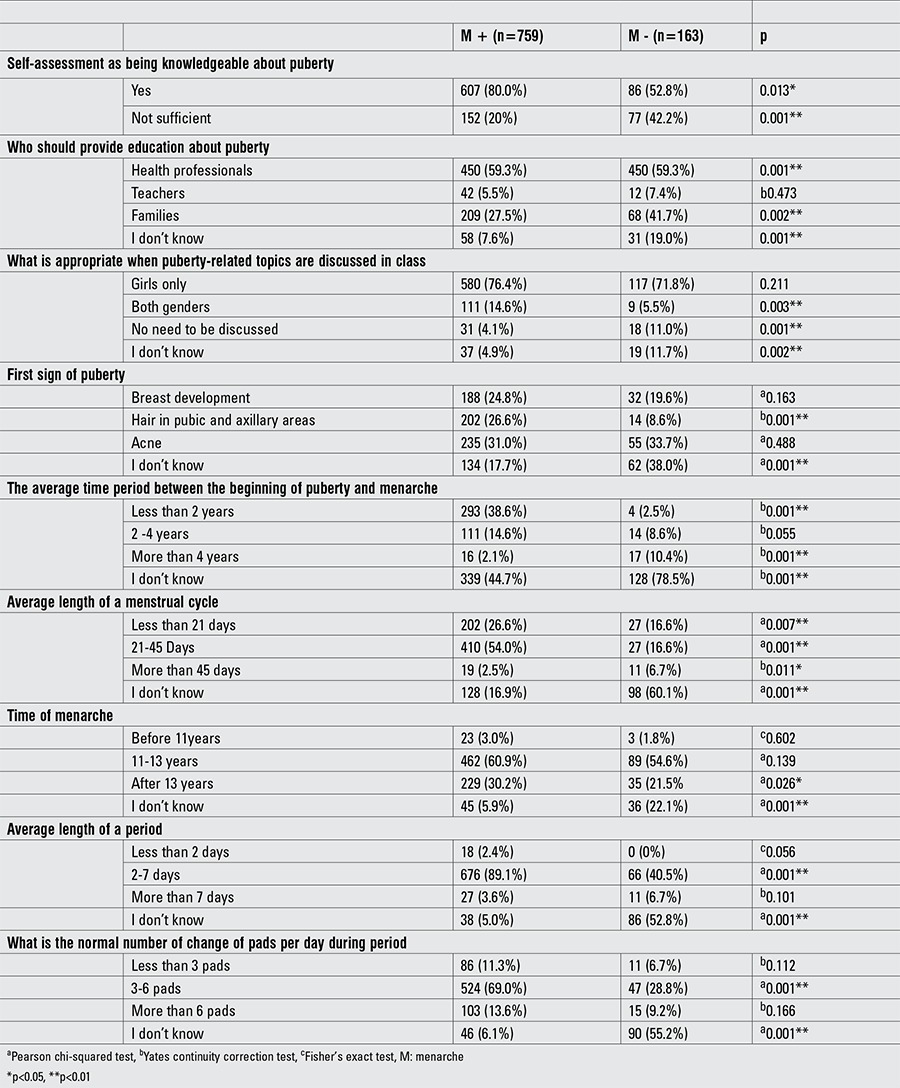
Evaluation about pubertal knowledge by menarche status
